# Influence of Salt Support Structures on Material Jetted Aluminum Parts

**DOI:** 10.3390/ma14154072

**Published:** 2021-07-21

**Authors:** Benedikt Kirchebner, Maximilian Ploetz, Christoph Rehekampff, Philipp Lechner, Wolfram Volk

**Affiliations:** 1 Chair of Metal Forming and Casting, Technical University of Munich, Walther-Meissner-Strasse 4, 85748 Garching, Germany; maximilian.ploetz@utg.de (M.P.); philipp.lechner@utg.de (P.L.); wolfram.volk@utg.de (W.V.); 2 Institute of Micro Technology and Medical Device Technology, Technical University of Munich, Boltzmannstrasse 15, 85748 Garching, Germany; christoph.rehekampff@tum.de

**Keywords:** additive manufacturing, material jetting, support structure

## Abstract

Like most additive manufacturing processes for metals, material jetting processes require support structures in order to attain full 3D capability. The support structures have to be removed in subsequent operations, which increases costs and slows down the manufacturing process. One approach to this issue is the use of water-soluble support structures made from salts that allow a fast and economic support removal. In this paper, we analyze the influence of salt support structures on material jetted aluminum parts. The salt is applied in its molten state, and because molten salts are typically corrosive substances, it is important to investigate the interaction between support and build material. Other characteristic properties of salts are high melting temperatures and low thermal conductivity, which could potentially lead to remelting of already printed structures and might influence the microstructure of aluminum that is printed on top of the salt due to low cooling rates. Three different sample geometries have been examined using optical microscopy, confocal laser scanning microscopy, energy-dispersive X-ray spectroscopy and micro-hardness testing. The results indicate that there is no distinct influence on the process with respect to remelting, micro-hardness and chemical reactions. However, a larger dendrite arm spacing is observed in aluminum that is printed on salt.

## 1. Introduction

Material jetting (MJT) additive manufacturing processes are based on the controlled droplet-wise deposition of build material. Commercially available MJT printers mainly focus on the processing of photopolymers and waxes [1]. However, the processing of molten metals [2] and molten salts [3] has also been demonstrated. Metal parts are of particular interest for industrial applications because in comparison with polymers, waxes and salts, they deliver high mechanical part strength. Perhaps the biggest advantage of additive manufacturing is the design freedom. Complex geometries and structures inclined up to a certain degree can be printed in metal MJT, as shown by Jayabal et al. [4], Sukhotskiy et al. [5] and Zhang et al. [6]. However, for full 3D-capability, most additive processes need some sort of support structure. The disadvantage of these support structures is that they have to be removed after the printing process, which increases costs and slows down the processing chain [7]. Therefore, the need for support structures should be minimized as far as possible, e.g., by varying the part’s orientation.

The support structure is often made of the same material as the part. In this case, the support structures can be optimized by the use of fine structures with low-volume fractions that reduce the effort required in machining processes, as shown by Hussein et al. [8]. Another approach is to make the support structure out of a different material than the part, which allows easier removal, e.g., by dissolution [9]. Soluble materials are already used in the foundry industry to make cores for high-pressure die casting [10]. Apart from pure salts [11], research has also been conducted in foundry cores made from salt mixtures [12]. Foundry cores are either squeezed, shot or cast [11]. Especially when handling salts in the liquid phase, i.e., in casting of the cores, the corrosiveness of the molten salts with respect to metals has to be considered [13]. Another characteristic of many salts is their low thermal conductivity [14]. All of these aspects also have to be considered when transferring the processing of molten salts from the foundry industry to additive manufacturing.

Previous investigations have focused on the design of an MJT print head and the selection of suitable salts for processing in an MJT process [3]. Suitable salt support structures withstand the temperatures of molten aluminum and demand far less rework than their metal counterparts. However, it is essential that the support material does not have significant negative effects on the printed part.

“How will the introduction of salt as support material influence our MJT process?” This is the research question we are aiming to answer by characterizing the interface of material jetted parts made of aluminum as a build material and salt as a support structure via optical microscopy, confocal laser scanning microscopy, energy-dispersive X-ray spectroscopy and micro-hardness testing. Similar methods are used by Shiran et al. to study the interface and inter-metallic compounds of samples made from aluminum and steel [15]. The question will be answered by searching for the presence of the following phenomena that we hypothesize to be indicators of a negative influence:

- Visual signs of corrosion at the aluminum–salt interface, especially where aluminum has come into contact with molten salt.
- Change in the surface structure due to remelting of the aluminum alloy in areas with molten salt contact.
- Salt residues on the aluminum surface after support removal and cleaning the surface.
- Larger dendrite arm spacing due to a slower solidification of aluminum on top of the support structure compared to the rest of the sample.
- Lower micro-hardness in areas where it was printed on salt because of the low thermal conductivity of salt and consequently slow solidification.

Our investigation is focused on the aluminum silicon alloy AlSi12(a) as build material and a eutectic mixture of potassium chloride (KCl) and sodium chloride (NaCl) as support material. Printing AlSi12(a) in an MJT process has already been demonstrated [16]. KCl-NaCl has been selected in previous work from a wide variety of salts and salt mixtures because of its comparatively good processability in a prototype MJT test stand [3]. Three different sample types are analyzed: aluminum printed on salt, salt printed on aluminum and aluminum printed directly on both the printing plate and the salt support.

## 2. Materials and Methods

### 2.1. Sample Description

#### 2.1.1. Build and Support Material

The build material for all samples is AlSi12(a) (4047A). It is a eutectic aluminum silicon alloy. The semi-finished product is a wire of 0.8 mm diameter (Drahtwerk Elisental W. Erdmann GmbH & Co, Neuenrade, Germany). The alloy has a solidus temperature of 577 °C and a liquidus temperature of 582 °C, as calculated using the METALS method described by Mills [17]. The alloy composition of a printed AlSi12(a) sample was analyzed with an optical emission spectrometer (FOUNDRY-MASTER, Worldwide Analytical Systems GmbH, Kleve, Germany). The five elements with the highest concentration in the alloy are listed in Table 1. Apart from silicon, the main alloying element is iron, with an average concentration of 0.134%.

**Figure 1:**
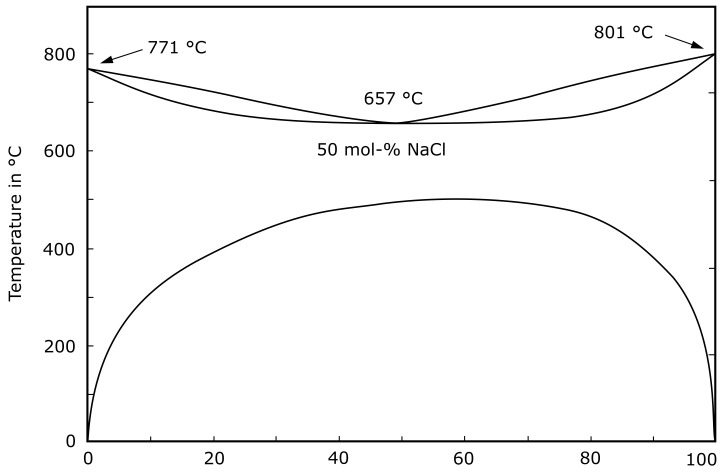
Phase diagram of KCl and NaCl according to Bale et al. [20]. The melting temperature of the eutectic mixture is 657 °C. There is a solid solution miscibility gap up to temperatures of approximately 500 °C [19].

The support material is a water-soluble salt mixture (KCl and NaCl). The two individual salts have ≥99.5% purity. They are mixed in powder form and fed into the print head, where they are dissolved and melted. Figure 1 shows the phase diagram of KCl and NaCl. At room temperature, the KCl-NaCl solutions are thermodynamically unstable and separate upon contact with small amounts of water [18]. Up to temperatures of approximately 500 °C, there is a miscibility gap and KCl-NaCl mixtures exist in two phases. A single solid solution phase is observable above the miscibility gap. The minimum liquidus temperature of the system is 657 °C (eutectic mixture) [19].

#### 2.1.2. Geometries

**Figure 2:**
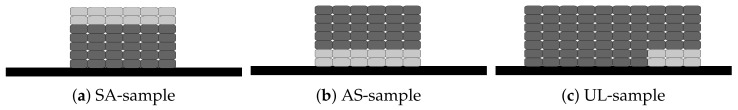
Schematic representation of the SA-sample (**a**), AS-sample (**b**) and UL-sample (**c**). Dark gray areas designate the aluminum part, light gray areas the support structure and black areas the heated nickel-plated steel printing plate.

For the analyses, three different sample geometries were examined. Firstly, a salt support structure with aluminum printed on top of it (AS-sample), secondly, an aluminum part with salt printed on top of it (SA-sample), and lastly, an aluminum part in the shape of an upside down letter “L” that is partly printed on a salt support structure and partly printed on the printing plate directly (UL-sample). The main difference between the different samples is that in the AS-sample, molten aluminum is printed on solidified salt, while in the SA-samples, the salt is in the molten phase when it comes into contact with the aluminum. The UL-sample is basically a modified AS-sample with parts of aluminum that are printed directly on the printing plate rather than onto the salt support structure. Table 2 and Figure 2 provide an overview of the sample geometries.

#### 2.1.3. Sample Preparation

**Figure 3:**
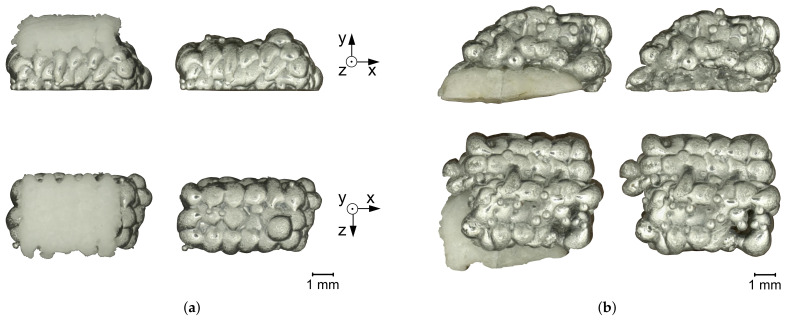
SA-sample (**a**) and AS-sample (**b**) with salt support structure and after support removal. The top row shows the samples viewed from the side, the bottom row viewed from above.

**Figure 4:**
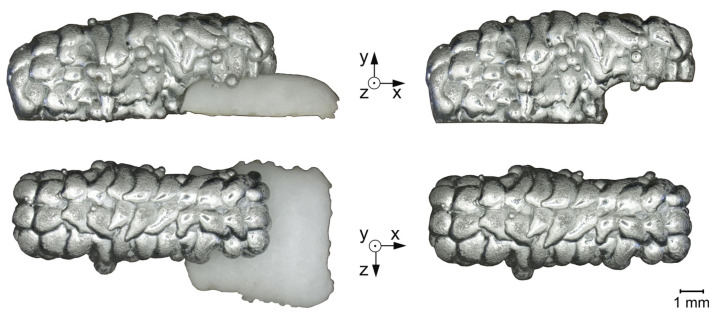
UL-sample with salt support structure and after support removal. The top row shows the sample viewed from the side, the bottom row viewed from above.

The samples are printed sequentially, e.g., for the AS-sample, the first layers are printed with a print head for aluminum. After changing the print head to the salt print head, the remaining layers are printed. Changing the print head interrupts the nitrogen purging. Nitrogen purging is necessary to prevent excessive oxidation and ensure good connection between the aluminum droplets. Since the nitrogen purging is interrupted only after all aluminum droplets are printed and solidified, the connection between the droplets in the samples is not influenced. The printed samples are depicted in Figure 3 and Figure 4. Of the three prepared UL-samples, only one representative part is shown.

### 2.2. Experimental Setup

There is a wide variety of methods for droplet ejection in metal MJT, including piezoelectric, magnetohydrodynamic, pneumatic, impact-driven and laser-induced [21]. All samples for this investigation are printed on a pneumatically actuated drop on-demand MJT test stand with interchangeable print heads, that was originally developed for printing aluminum. The test stand is described in detail by Himmel et al. [16]. For salt printing, a print head was designed with materials that were selected in a previous work [3]. Droplets are ejected by a pressure surge and fall onto a heated printing plate, which is a nickel-plated steel plate. The print head is stationary and the printing plate moves under G-code control inside a nitrogen-purged printing chamber. The nitrogen purging is necessary to prevent excessive oxidation of the droplets and ensure good connection between the droplets.

A Zeiss Axioplan 2 with an AxioCam Mrc5 (Carl Zeiss Microscopy Deutschland GmbH, Oberkochen, Germany) was used for optical microscopy. Confocal laser scanning microscopy was carried out using a VK-X100 series 3D laser scanning microscope (KEYENCE DEUTSCHLAND GmbH, Neu-Isenburg, Germany). A JSM-7500F scanning electron microscope (JEOL (Germany) GmbH, Freising, Germany) with a 50 mm${}^{2}$ X-MAX-Detector and the software INCA^®^ (Version 4.15, 2009, Oxford Instruments plc, Abingdon, UK) was used for the energy-dispersive X-ray spectroscopy. The micro-hardness tests were performed on a LM100AT micro-hardness tester (LECO Instrumente GmbH, Mönchengladbach, Germany).

### 2.3. Experimental Procedure

**Figure 5:**
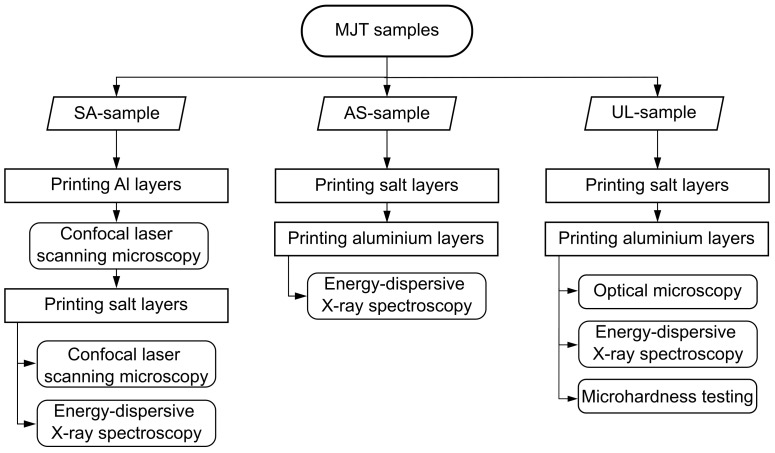
Flow chart of the experimental procedure. Three sample geometries (SA-sample, AS-sample and UL-sample) are printed out of aluminum and salt via Material Jetting (MJT). For the SA-sample, the aluminum layer is printed first and then analyzed using confocal laser scanning microscopy. Confocal laser scanning microscopy is performed again on the aluminum layer after it has been printed with molten salt. Then, energy-dispersive X-ray spectroscopy is performed. For the AS-sample, the salt and aluminum layers are printed successively and the aluminum surface that came into contact with salt is analyzed using energy-dispersive X-ray spectroscopy. All layers of the UL-samples are also printed successively. Optical microscopy, energy-dispersive X-ray spectroscopy and micro-hardness testing are performed.

All three samples follow different preparation and analysis procedures. For the SA-sample, the aluminum layer is printed first and then analyzed using confocal laser scanning microscopy to determine the surface structure. Next, the salt layer is printed. After removal of the salt layer, the aluminum surface is once again analyzed using confocal laser scanning microscopy to determine if there have been any changes in the surface structure. Finally, energy-dispersive X-ray spectroscopy is performed on the cleaned sample to determine if there are salt residues which might be due to chemical reaction. For the AS-sample, the salt and aluminum layers are printed successively. The salt is removed and the aluminum surface that came into contact with salt is analyzed using energy-dispersive X-ray spectroscopy after having been cleaned. All layers of the UL-samples are also printed successively. After removing the salt, the samples are cold-mounted. Optical microscopy, confocal laser scanning microscopy, energy-dispersive X-ray spectroscopy and micro-hardness testing are performed. A flow chart of the experimental procedure is shown in Figure 5.

#### 2.3.1. Optical Microscopy

**Figure 6:**
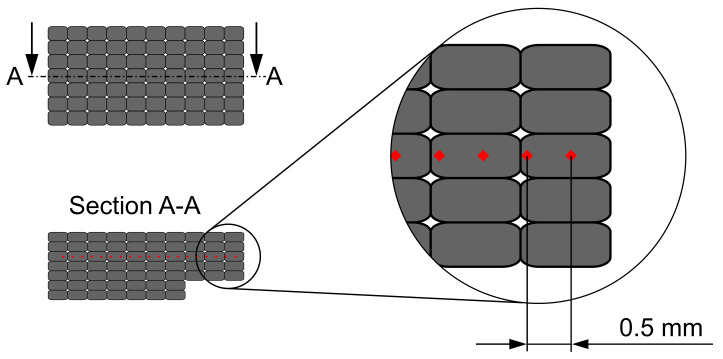
Measuring grid for micro-hardness measurement. The measuring points are located in the center of the sample in the vertical direction so that the hardness measurement is not distorted by possible edge influences. In the horizontal direction, the distance between the measuring points and the samples’ edges is maintained in accordance with DIN EN ISO 6507-1. The distance between the measuring points is 0.5 mm.

In order to investigate the influence of the support structure on the formation of the material microstructure and to determine if there are visual signs of corrosion, optical microscopy of the UL-sample surfaces was performed. Optical microscopy was conducted for three samples. Before optical microscopy, the salt support structure is removed and the aluminum samples are cleaned in acetone to remove salt residues and impurities on the sample’s surface. The cleaned specimens are then cold-mounted in epoxy resin (Kulzer GmbH, Hanau, Germany). The mounted UL-samples are ground down to section A-A (see Figure 6) so that the optical inspection can be performed in the center of the sample. This position was chosen because the microstructure at this location is less affected by possible edge effects. Following grinding, the surface of the specimen is polished. The sample’s surface is etched in order to make the material microstructure visible for its assessment. To investigate the microstructure, the samples are etched in two different ways. The first etching variant is a single etching with two percent aqueous sodium hydroxide solution, which acts for 60 s. In the second etching variant, a double etching process is performed. In a first step, the polished surface is treated with two percent aqueous sodium hydroxide solution for 60 s. This is followed by a further treatment of the surface with alkaline potassium permanganate solution according to Weck et al. [22] for 15 s. Finally, optical microscopy is performed.

#### 2.3.2. Energy-Dispersive X-ray Spectroscopy

In energy-dispersive X-ray spectroscopy, an electron beam is focused on the sample, resulting in electrons being ejected from the atoms of the sample. Electrons from higher shells fill the vacancies, which ultimately results in the emission of X-rays that are characteristic of the atom, thus enabling an identification. The X-rays can be designated according to the Siegbahn notation, which denotes the electron transition using a Latin and a Greek letter as well as an Arabic number. The Latin letter designates the destination shell of the transitioning electron (K,L,M) and the Greek letter its origin in relative terms. The number further specifies the originating sub-shell [23].

K${}_{\alpha 1}$, for example, denotes the X-ray that was emitted due to an electron transition to the K-shell from the L${}_{\mathrm{III}}$-sub-shell. L${}_{\eta }$ denotes the X-ray that was emitted due to an electron transition to the L${}_{\mathrm{II}}$-sub-shell from the M${}_{I}$-sub-shell [24].

All three sample types were analyzed using energy-dispersive X-ray spectroscopy. For both the SA- and AS-sample, the aluminum surface that came into contact with salt was analyzed in three locations. One UL-sample was analyzed in the cross-section at three locations. To prepare the samples for energy-dispersive X-ray spectroscopy, for the SA- and AS-sample, the salt support structure is first manually removed using steel tweezers. Then, the samples are immersed in an ultrasonic bath of distilled water for one minute to remove more of the salt. Afterwards, they are dried with a blow dryer. Finally, the samples are cleaned by immersing in ethanol for one minute and again dried with a blow dryer. For the analyses of the UL-sample, the cold-mounted samples from the optical microscopy were used. The energy-dispersive X-ray spectroscopy-mappings are taken with an accelerating voltage of 10 kV. Any signal from 0 to 0.15 keV is ignored to cut off the noise peak.

#### 2.3.3. Confocal Laser Scanning Microscopy

With confocal laser scanning microscopy, it is possible to reconstruct 3D-structures in the micrometer scale, thus allowing an examination of the surface structure. Confocal laser scanning microscopy was performed on the SA-sample. The surface of three droplets is scanned before the salt layer is applied and afterwards. We look for changes in the surface structure due to remelting of the aluminum that might occur due to heat transfer from the molten salt.

#### 2.3.4. Micro-Hardness Testing

To determine the influence of the support structure on the mechanical material properties, micro-hardness tests were carried out based on the Vickers method. The test load is HV0.025 (24.52 mN). The micro-hardness was investigated using three UL-samples, which were also examined using optical microscopy. Before the micro-hardness measurements are performed, the samples are cold-mounted. The samples’ surfaces used for the micro-hardness measurement are prepared in accordance with DIN EN ISO 6507-1. For the measurement grid, a line is selected for all three samples on which the individual measurement points are located. Figure 6 shows the measurement grid used.

The measuring points are located in the center of the sample so that the micro-hardness measurement is not distorted by possible edge influences. The vertical distance of the measuring points from the edges of the samples is chosen in such a way that the measuring points located on the measuring line are at approximately the same distance from the upper and lower edges of the area that was printed on salt. The distance between the measuring points is 0.5 mm. In the horizontal direction, a distance of at least three times the average indentation diagonal length according to DIN EN ISO 6507-1 is maintained from the edge of the sample. The individual UL-samples differ slightly in size. Depending on the sample’s length, 20 to 22 measuring points are defined on the sample’s surfaces, 7 to 9 points are located in the sample’s area that is printed on salt and the remaining points are located in the area that is printed on aluminum.

## 3. Results and Discussion

### 3.1. Optical Microscopy

**Figure 7:**
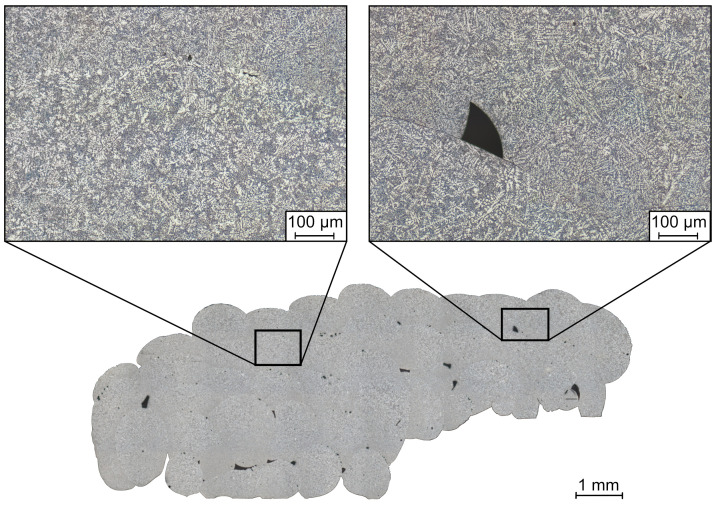
Cross-sectional area of UL-sample etched with two percent aqueous sodium hydroxide solution. The images above the cross-sectional area show the detailed views of the material microstructure of the sample area above the aluminum (left side) and above the salt support structure (right side). In the area above the salt support structure, a coarser dendrite structure can be observed.

**Figure 8:**
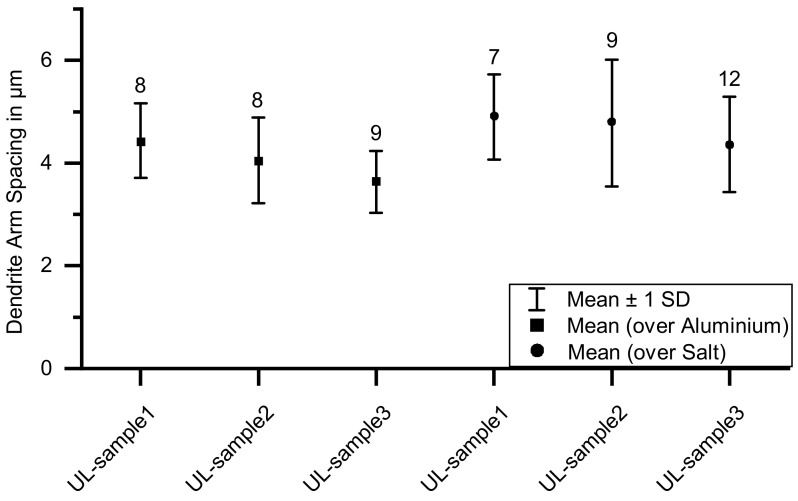
Dendrite arm spacing in the aluminum part (UL-sample). The squares show the dendrite arm spacing in the area above the aluminum and the circles show the dendrite arm spacing above the salt support structure. The numbers above the result points show the number of measurements performed. Based on the measurements, a larger dendrite arm spacing tends to be observed in the area above the salt support structure.

In Figure 7 the cross-sectional area of a representative UL-sample etched with two percent aqueous sodium hydroxide solution is shown. Detailed views of the aluminum microstructure are shown above the sample section. On the left side is the material structure of the sample’s area printed on aluminum, and on the right, the area printed on salt. There are no clear visual signs of corrosion at the aluminum–salt interface where aluminum came into contact with salt. The needle-like structures appearing white due to the etching are dendrites. When comparing the detailed views, it can be seen that the dendrite structure is finer in the left area of the sample than in the right area. The salt support structure tends to coarsen the sample’s microstructure. As in previous investigations, the microstructure also changes within a droplet. At the bottom of a droplet, a finer microstructure can be observed than that at the top of the droplet [16]. The droplets tend to have a coarser dendrite structure in the region above the salt support structure. The gradient within a droplet is maintained. In the area above the salt support structure, the average dendrite arm spacing across all three UL-samples is 4.68 μm, and above the aluminum, 4.04 μm. Figure 8 shows the averaged dendrite arm spacing in the area above the aluminum and the salt support structure for the three samples investigated. The squares on the left side of the diagram describe the area above the aluminum and the circles to the right describe the dendrite arm spacing of the area above the salt support structure. The number of measurements is listed above the result points. The dendrite arm spacing varies slightly between the individual UL-samples. However, the tendency of the larger dendrite arm spacing in the region above the salt support structure can be observed for all three samples.

**Figure 9:**
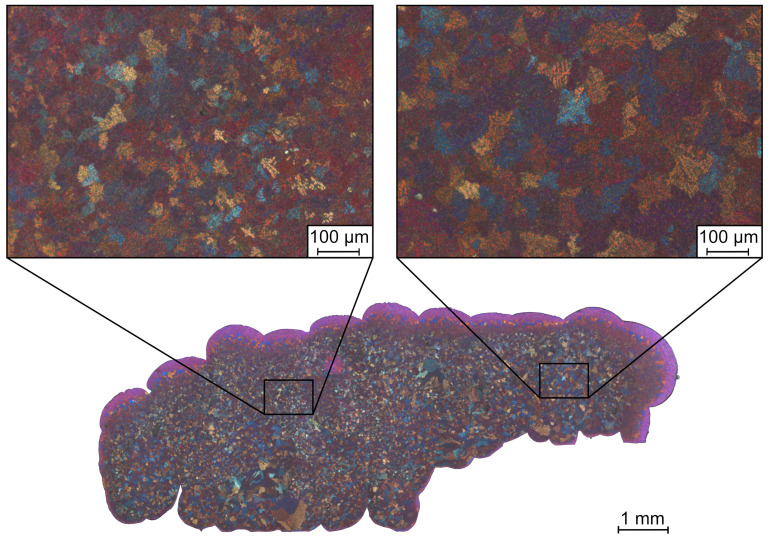
Cross-sectional area of UL-sample etched with two percent aqueous sodium hydroxide solution and alkaline potassium permanganate solution according to Weck et al. [22]. The images above the cross-sectional area show the detailed views of the material microstructure of the sample area above the aluminum (left side) and above the salt support structure (right side). A coarser dendrite structure can be seen in the area above the salt support structure.

The observation of the coarsening of the material structure can also be seen from the optical examination of the double-etched sample surface. Figure 9 shows the cross-sectional area of the double-etched UL-sample. In this representation, too, a coarser material structure can be seen in the area of the UL-sample printed over the salt support structure when compared to the area printed on aluminum. The presumed reason for the coarser microstructure in the area of the salt support structure is a slower cooling rate of the substrate as a result of the lower thermal conductivity of the salt support structure compared to aluminum. Each newly deposited droplet introduces thermal energy into the substrate. In the left region of the sample, the substrate is aluminum, which has better thermal conductivity than when the substrate is salt, as shown on the right. Consequently, the solidification of the aluminum droplets on salt is slower.

### 3.2. Energy-Dispersive X-Ray Spectroscopy

**Figure 10:**
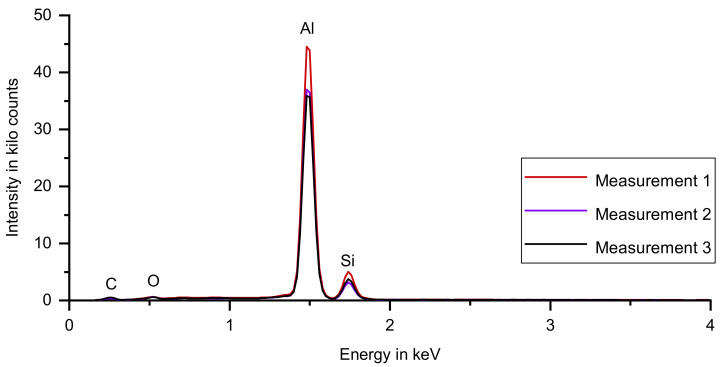
Three spectra for the SA-sample, superimposed in a single diagram. Aluminum, silicon, carbon and oxygen were detected. The three spectra differ in peak height, the peak positions do not change.

In Figure 10, the spectra for the SA-sample are depicted, superimposed in a single diagram. Aluminum, silicon, carbon and oxygen were detected. The presence of aluminum and silicon is trivial. Aluminum surfaces oxidize when exposed to the atmosphere, offering a possible explanation for the detection of oxygen. The carbon signal might stem from the sample preparation with a carbon-containing tape to ensure good electrical conductance of the sample, which is crucial for energy-dispersive X-ray spectroscopy. The three spectra differ in peak height, which possibly originates from different measuring durations. More importantly, however, the peak positions do not change.

Potassium and carbon share characteristic X-rays of similar energies, e.g., carbon K${}_{\alpha }$ at approximately 0.28 keV and potassium L${}_{\eta }$ at approximately 0.26 keV [25]. To differentiate between these two elements, the inspection of other characteristic X-rays is advisable. Potassium has a K${}_{\alpha 1}$ peak at approximately 3.3 keV. The inspected samples exhibit very low signal power at this energy level so the identification of carbon instead of potassium seams appropriate.

**Figure 11:**
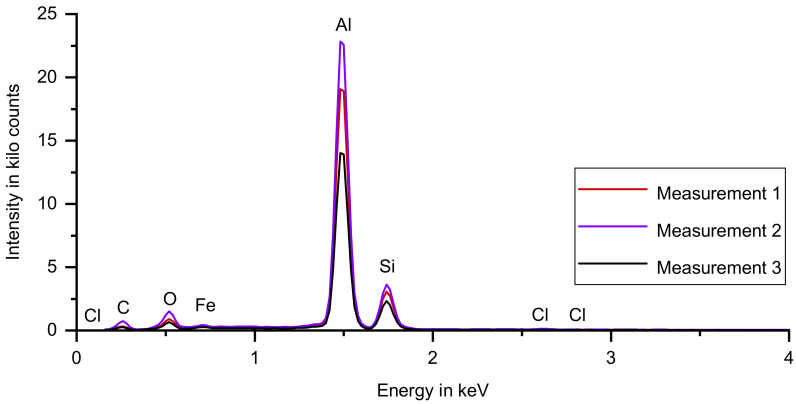
Three spectra for the AS-sample, superimposed in a single diagram. Aluminum, silicon, carbon, oxygen, iron and chlorine were detected. The three spectra differ in peak height, the peak positions do not change. One of the three spectra shows a weak chlorine signal with all peaks < 150 counts.

Figure 11 shows the spectra of the AS-sample. In contrast to the results of the SA-sample, in all three spectra, iron is detected, which is a minor alloying element of AlSi12(a). All peak positions do not change for the three individual measurements. One of the three spectra shows a weak chlorine signal with all peaks < 150 counts. Chlorine is likely to stem from the salt support structure which is constituted by two chlorine salts. Distinguishing between chlorine-containing corrosion products and insufficient removed support material is not possible in energy-dispersive X-ray spectroscopy. However, the results of the optical microscopy suggest that the chlorine does not stem from a corrosion product.

**Figure 12:**
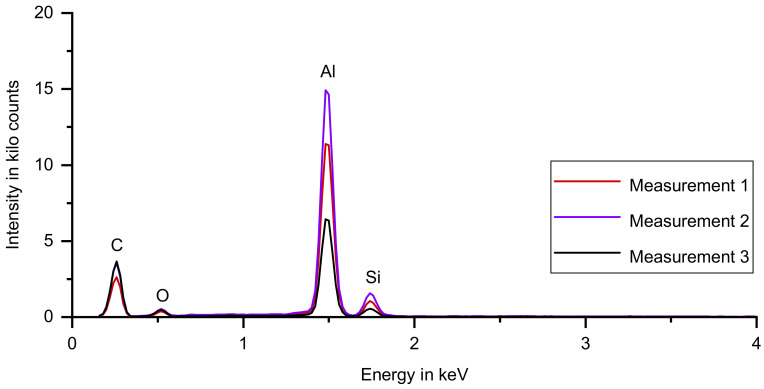
Three spectra for the UL-sample, superimposed in a single diagram. Aluminum, silicon, carbon and oxygen were detected. The three spectra differ in peak height, the peak positions do not change.

Figure 12 shows the spectra of the UL-sample, which is qualitatively identical to the results of measurements of the SA-sample.

Sodium or potassium were not detectable in any sample in energy-dispersive X-ray spectroscopy.

### 3.3. Confocal Laser Scanning Microscopy

**Figure 13:**
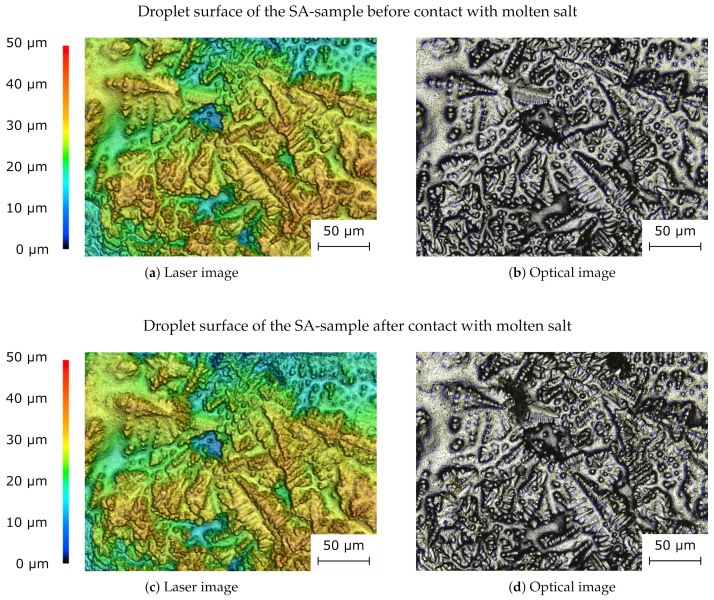
Surface of the SA-sample analyzed with the confocal laser scanning microscope. The top images show the sample’s surface before coming into contact with molten salt and the bottom two images show the surface after molten salt contact. Next to the laser images (**a**,**c**), the corresponding optical images are displayed (**b**,**d**). No significant change in the surface can be seen. The results of the confocal laser scanning microscopy for the other two droplets do not differ qualitatively.

The result of the confocal laser scanning microscopy for one representative droplet is shown in Figure 13. Subfigure (a) shows the laser scan of the sample before it has been exposed to molten salt, and subfigure (b) shows an optical image of the sample. Subfigures (c) and (d) show the samples after molten salt exposure. Dendrites are visible on the surface. No significant change of the surface structure can be seen. If remelting had occurred, a change in the surface should be noticeable since it is unlikely that dendritic structures would form again in exactly the same way after remelting. The results of the confocal laser scanning microscopy for the other two droplets do not differ qualitatively.

### 3.4. Micro-Hardness Testing

**Figure 14:**
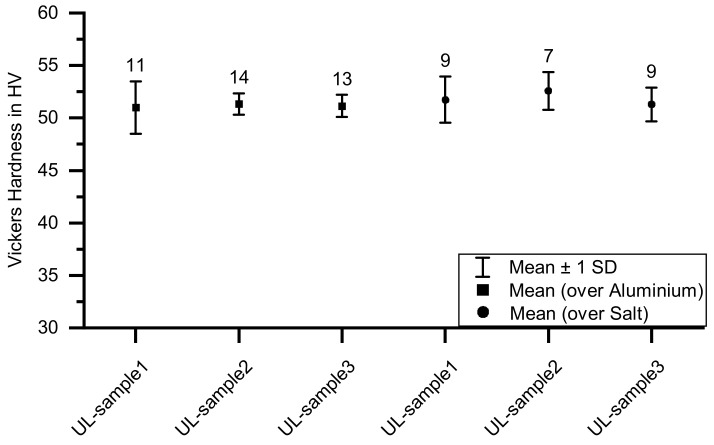
Vickers micro-hardness values in the aluminum part (UL-sample). The squares show the micro-hardness values in the area above the aluminum and the circles show the micro-hardness values above the salt support structure. The numbers above the result points show the number of measurements performed. Based on the measurements, no significant influence of the salt support structure on the micro-hardness occurring in the aluminum can be determined.

Figure 14 shows a comparison of the material micro-hardness of the sample’s area printed on aluminum and the area printed on the salt support structure. The squares on the left side of the diagram describe the area above the aluminum and the circles to the right describe the micro-hardness of the area above the salt support structure. The number of measurements is listed above the result points. All three UL-samples are used to investigate the material micro-hardness.

Despite different dendrite arm spacing, there is no discernible micro-hardness gradient. The mean of the material micro-hardness for all six measuring ranges is between 51.0 and 52.6 HV0.025. The scatter bands of the material micro-hardness of the areas above the salt support structure are somewhat larger, with a slight tendency towards higher values. In summary, there is no significant change in material micro-hardness.

## 4. Conclusions

In this article, material jetted aluminum samples with salt support structure were analyzed. Optical microscopy, confocal laser scanning microscopy, energy-dispersive X-ray spectroscopy and micro-hardness tests were conducted to determine the effect of the salt support structures on the aluminum parts. There were no clear visual signs of corrosion at the aluminum–salt interface where aluminum came into contact with molten salt, and no remelting was observed. In one sample, some residue of the salt’s components was detectable on the aluminum surface via energy dispersive X-ray spectroscopy. There was no significant change in the micro-hardness of aluminum that was printed on aluminum compared to aluminum that was printed on salt. Coming back to our research question—“How will the introduction of salt as support material influence our MJT process?”—our answer is: There is no distinct negative influence on the process, however, minor changes in the aluminum microstructure are observable. The results of this study indicate that KCl-NaCl might constitute a suitable water-soluble support material for material jetted aluminum parts. We showed how salt support structures influence an aluminum MJT process with simple part geometries. For more complex part geometries and larger support structures, further research is necessary to evaluate the effects of the salt support structure on the process. 

## Figures and Tables

**Table 1:** Composition of a material jetted AlSi12(a) sample as determined by optical emission spectroscopy. All values in %.

Element	Average Concentration	Concentration in Measurement 1	Concentration in Measurement 2	Concentration in Measurement 3
Al	86.6	86.6	86.6	86.6
Si	13.1	13.0	13.1	13.1
Fe	0.134	0.137	0.128	0.136
Sr	0.022	0.027	0.019	0.021
Ti	0.018	0.018	0.017	0.018

**Table 2:** Sample geometries.

Description	Abbreviation
Salt printed on aluminum	SA-sample
Aluminum printed on salt	AS-sample
Aluminum printed both on salt and on printing plate	UL-sample

## Data Availability

All the data is available within the manuscript.

## Abbreviations

The following abbreviations were used in this manuscript:

3D	Three-dimensional
Al	Aluminum
AS-sample	Aluminum part with salt printed on top
Fe	Iron
KCl	Potassium chloride
MJT	Material jetting
NaCl	Sodium chloride
SA-sample	Salt support structure with aluminum printed on top
Si	Silicon
Sr	Strontium
Ti	Titanium
UL-sample	Aluminum part in the shape of an upside down letter “L”, partly printed on salt support structure
